# Trajectories of cognitive function among people aged 45 years and older living with diabetes in China: Results from a nationally representative longitudinal study (2011~2018)

**DOI:** 10.1371/journal.pone.0299316

**Published:** 2024-05-24

**Authors:** Shi Chen, Yuewei Ling, Faquan Zhou, Xu Qiao, Jan D. Reinhardt

**Affiliations:** 1 Institute for Disaster Management and Reconstruction of Sichuan University and Hong Kong Polytechnic University, Sichuan University, Chengdu, China; 2 West China School of Public Health and West China Fourth Hospital, Sichuan University, Chengdu, China; 3 Department of Management Science and Engineering, Stanford University, Stanford, CA, United States of America; 4 Department of Vascular Surgery, First Affiliated Hospital, Army Medical University (Third Military Medical University), Chongqing, China; 5 Department of Health Sciences and Medicine, University of Lucerne, Lucerne, Switzerland; 6 Rehabilitation Medicine Centre, Jiangsu Province Hospital/Nanjing Medical University First Affiliated Hospital, Nanjing, China; Universita Cattolica del Sacro Cuore Sede di Roma, ITALY

## Abstract

**Objectives:**

Diabetes is associated with decline of cognitive function. Exploring different trajectories of cognitive function occurring in people with diabetes is important to improved prognosis. This study aimed to investigate differential patterns of trajectories of cognitive function and baseline determinants of trajectory group membership utilizing data from middle-aged and older Chinese adults with diabetes.

**Methods:**

Participants of the Chinese Health And Retirement Longitudinal Study (CHARLS) aged 45 years and above received biennial assessments between 2011 and 2018. The primary outcome was overall cognitive function score operationalized as sum of mental intactness and episodic memory scores derived from the Telephone Interview of Cognitive Status (TICS). A weighted growth mixture model was used to estimate cognitive function trajectories of CHARLS participants with diabetes, and baseline factors associated with trajectory group membership were investigated with weighted multinomial logistic regression.

**Results:**

Data from 1,463 participants with diabetes aged 45 years and above were analyzed, a three-group trajectory model showed the best fit for overall cognitive scores: low baseline, linear declining (22.1%); moderate baseline, linear declining (37.5%) and high-stable (40.3%). Older participants, females, participants with low education, with nighttime sleep <6 h, without daytime napping habits, and with depressive symptoms were at a higher risk of unfavorable cognitive function trajectories.

**Conclusions:**

We identified heterogeneous trajectories of cognitive function among middle-aged and older people living with diabetes in China. Socially vulnerable groups including females, rural residents, and those with low education were at a higher risk for unfavorable trajectories. In health programs aimed at preventing and mitigating cognitive decline in individuals with diabetes more attention should be given to vulnerable groups. Reduced nighttime sleep, lack of daytime napping, and depressive symptoms appear to be modifiable risk factors.

## Introduction

Prevalence of diabetes increases with demographic aging of populations [1–3]. In China, a recent nationally representative cross‐sectional study from 2021 reported that an estimated 13.21% of adults aged 45 years or older live with diabetes [4]. Previous studies have shown that diabetes is associated with lower cognitive function, concluding that cognitive dysfunction is an important comorbidity in diabetes [5, 6]. Diabetes is a major risk factor of cognitive decline, as hyperglycemia-mediated advanced glycation end-products(AGE) production, together with oxidative stress, are regarded as factors that can degenerate neurons and damage the vascular endothelium leading to impairment in cognitive function [7]. Recent evidence shows a direct association between accumulation of AGEs and the development of diabetic vascular complications [7]. **Diabetes conferrs a 1.25- to 1.91-fold excess risk for cognitive disorders (cognitive impairment and dementia) [8].** Decreased cognitive function is of concern because it results in poor quality of life, disability, as well as increased mortality risk and healthcare costs [6, 9]. Importantly, cognitive dysfunction is further associated with poor diabetes treatment adherence, resulting in health-related complications [10–12]. Studies point out that factors such as age, education, alcohol consumption, smoking, and depression are associated with cognitive trajectories [9], but evidence in individuals with diabetes is lacking. Given a large population of individuals with diabetes in China, exploring cognitive trajectories in order to identify potentially modifiable baseline risk factors for cognitive decline is critical for prevention and early management [13, 14]. Therefore, this study aimed to explore differential patterns of cognitive function trajectories and baseline risk factors for cognitive decline among middle-aged and older Chinese adults with diabetes.

## Methods

### Design

Prospective cohort study.

### Setting

This study utilizes data from **The China Health and Retirement Longitudinal Study (**CHARLS). CHARLS is a nationally representative study that aims to support scientific research on the Chinese population aged ≥ 45 years [15]. A four-stage, stratified, cluster random sampling method was adopted to recruit CHARLS participants and ensure national representativeness of the sample. The baseline survey was conducted in 2011 (wave 1), with 17,708 respondents from 150 counties of 28 Chinese provinces. Data were collected face-to-face with computer-assisted personal interviews [15]. Follow-up surveys with publically available data considered here were conducted in 2013 (wave 2), 2015 (wave 3), and 2018 (wave 4) [15]. More Details on the CHARLS are available elsewhere [15]. All participants of CHARLS provided written informed consent and the study protocol was approved by the Ethics Review Committee of Peking University (IRB00001052-11015).

### Assessment of diabetes

In this study, diabetes was operationalized using baseline information. One of the following conditions needed to be fulfilled so as to classify a participant as having diabetes: 1) history of taking medicines for diabetes (insulin injections or other traditional Chinese or modern medicines), 2) fasting plasma glucose (FPG) ≥126 mg/dl measured after at least 8 hours of fasting, or 3) glycosylated hemoglobin (HbA1c) ≥6.5% [4, 16]. In the CHARLS, FPG, and HbA1c were assessed using an enzymatic colormetric test and Boronate affinity HPLC 1, respectively.

### Study population

Inclusion criteria were as follows: (1) Diagnosed with diabetes at baseline (as mentioned in the section on assessment of diabetes); (2) Without self-reported doctor-diagnosed cognitive-related disease (Alzheimer’s disease, brain atrophy and Parkinsonism) at baseline. Exclusion criteria were: (1) No baseline cognitive function data; (2) Less than two follow-up measurement points for which cognitive function data were available.

**Fig 1:**
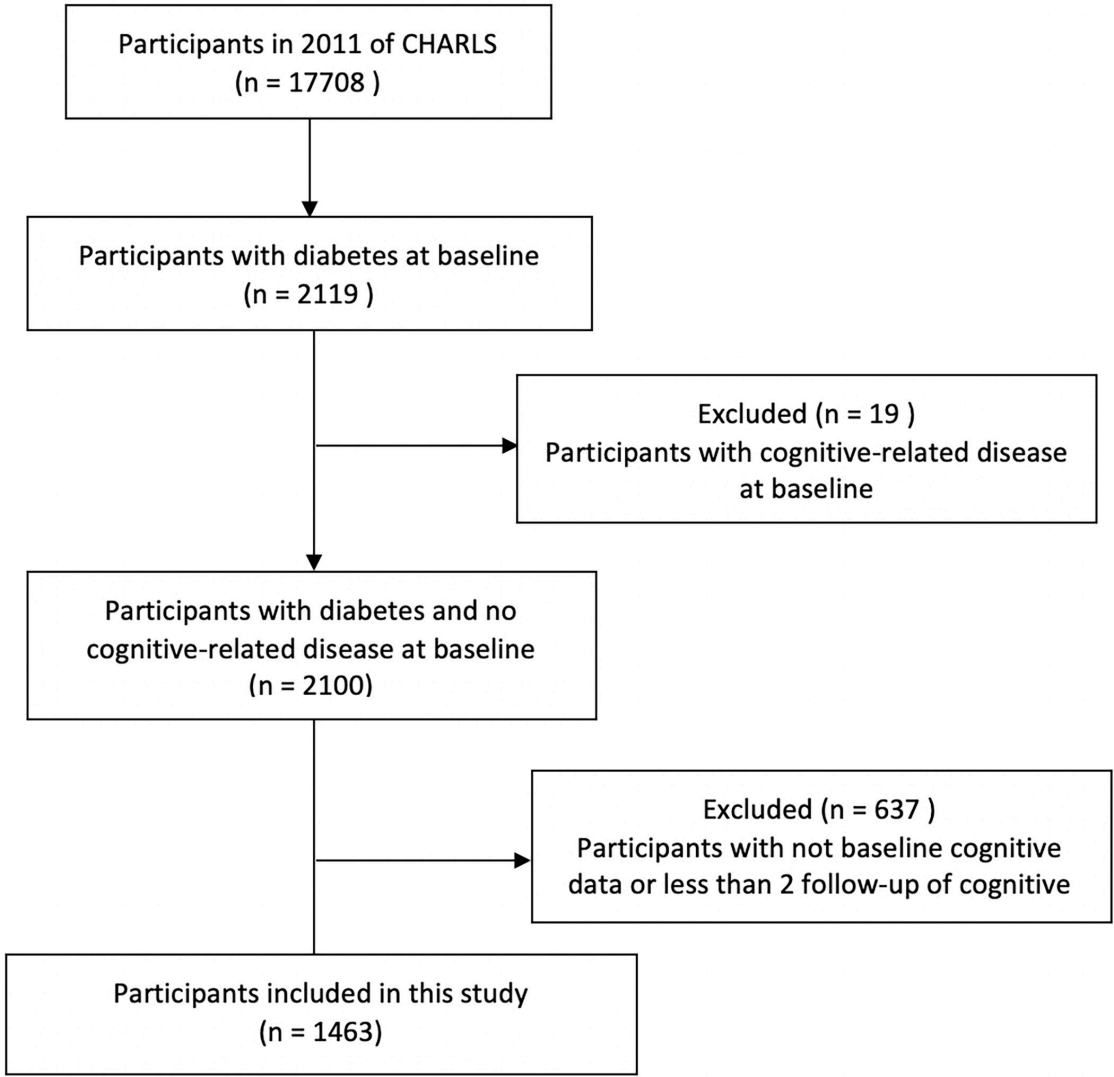
Study flow chart.

Of 17,708 participants interviewed in the 2011 CHARLS baseline survey, 2,119 had indications of diabetes at baseline. We excluded 19 participants with cognitive impairments and diseases at baseline. An additional 637 participants were excluded as baseline cognitive function data were not available or data from less than two follow-up measurement points had been collected. Eventually, data from 1,463 participants were analyzed (Fig 1). Of these, data were available at follow-up in 2013 for 1458, in 2015 for 1446, and in 2018 for 1439 participants.

### Cognitive function

Following previous studies using CHARLS data [14, 17], cognitive function was operationalized along two dimensions: mental intactness and episodic memory. Mental intactness was assessed with selected items of the Telephone Interview of Cognitive Status (TICS) battery designed to capture the cognitive state and screen for cognitive impairment of individuals [18]. Specifically, contents of the mental intactness assessment included stating current date (month, day, and year), the day of the week, the season of the year, a picture redrawing test, and serial subtracting 7 from 100 (5 times). Scores range from 0 to 11 with higher scores indicating better mental intactness. Episodic memory was evaluated with immediate and delayed recall of words. Participants were asked to recall as many words as possible immediately after the examiners read a list of 10 random words. Subsequently, participants were instructed to recall these words again ten minutes later. Each correctly recalled word was scored 1 so that the total score for immediate and delayed word recall ranged from 0 to 10 each. Total episodic memory scores were then determined as arithmetic average of immediate and delayed word recall, again ranging from 0 to 10. Finally, an overall cognitive function score was calculated as the sum of the scores of mental intactness and episodic memory, ranging from 0 to 21 with higher scores indicating better cognitive function [14].

### Baseline risk factors

Traditional methods of adjusting for confounding may miss or even introduce bias, which can be addressed by using a directed acyclic graph (DAG) to identify relevant confounders to be included in regression models [19]. Here, potential baseline factors were selected based on a DAG reported in a previous study [19]. Baseline survey information on age, gender, education level, smoking, alcohol consumption, nighttime sleep, daytime napping, and depressive symptoms were included as potential determinants of cognitive trajectory. Age was classified as “45~59,” “60~74,” and “≥75”. Education level included “no formal education”, “primary school”, “middle school or above”. Smoking was classified into “life-time non-smoker”, “current smoker”, or “former smoker”. Frequency of drinking alcohol in the last year was classified as “none,” “<once a month”, and “≥once a month”. Nighttime sleep was reported as “<6 h”, “6- 8h”, and “≥8 h”. Daytime napping was reported as “not at all”, “1–60 min”, and “>60 min”. Depressive symptoms were assessed using the Chinese version of the 10-item Center for Epidemiologic Studies Depression Scale (CESD-10), with the total score ranging from 0 to 30 and higher scores indicating more depressive symptoms. According to a previously established threshold, possible clinically relevant depression was defined as CESD-10≥ 10 [20].

### Statistical analysis

Characteristics of the study participants at baseline were summarized using mean ± standard deviation for continuous variables and percentages for categorical variables.

In models, we used 2011 sampling weights provided by the CHARLS team to account for multistage sampling. In weighted trajectory analysis, the primary outcome was overall cognitive function score, and secondary outcomes were mental intactness and episodic memory scores.

First, we utilized growth curve modeling (GCM) to identify the model with the best growth parameters. The entire sample was treated as a cohesive and homogeneous unit. We evaluated three growth parameters: an intercept-only model (no growth), an intercept-and-slope model (linear growth), and an intercept, linear parameter, and quadratic parameter model (nonlinear growth). Evaluation matrices included Tucker–Lewis Index (TLI), comparative fit index (CFI), and root mean square error of approximation (RMSEA). The growth parameter associated with the model exhibiting the most favorable goodness of fit was selected and then extended to growth mixture modeling (GMM).

Next, we employed GMM to categorize individuals into distinct groups exhibiting similar cognitive trajectories over time. To capture latent classes within the sample, GMM introduced a latent categorical variable, referred to as "class," which allowed for variability in growth parameters both between and within each identified class. Model selection involved determining the optimal number of groups that best characterized the data. To this end, we let the number of classes vary from 1 to 5 to identify the most suitable number of latent trajectory groups. Model fit statistics, including Akaike’s Information Criterion (AIC), Bayesian Information Criterion (BIC), Sample Size-Adjusted Bayesian Information Criterion (saBIC), entropy, and Lo-Mendell-Rubin adjusted likelihood ratio test (ALRT) were employed to evaluate the fit of GMMs with different numbers of classes. Smaller values of AIC, BIC, and saBIC indicated better model fit [21, 22]. As, however, in large datasets with numerous indicators, an increase in the number of classes can lead to a consistent decrease in these indices, favoring more complex models, elbow plots were also utilized to identify points of inflection or plateauing in the information criteria [23]. Furthermore, ALRT compared the fit of the current model with the previously estimated model having one class less, with a p-value<0.05 considered as indication that the preceding model ought to be preferred. Entropy values denote better class separation, it was noted that an over-fit model might also result in high entropy [23]. To account for shortfalls of individual fit indices and ensure a comprehensive assessment, all above goodness of fit measures were considered alongside investigators’ judgments of theoretical and practical coherence, in order to identify a useful and parsimonious GMM.

Chi-square test for categorical variables and ANOVA for continuous variables were next used to test differences among identified cognitive trajectory groups.

A weighted multinomial logit model then estimated the association of above-specified baseline risk factors with overall cognitive function trajectories of the best-fitting trajectory model. Adjusted odds ratios (OR) and corresponding 95% confidence intervals (CI) estimated with this multinomial logit model adjusted for confounders identified by DAGs reported in previous studies are provided [19, 24]. Statistical methods used for the secondary outcomes mental intactness and episodic memory are the same as above. For additional details, see S3 and S4 Tables.

Missing values at baseline (educational level 1, nighttime sleep 11, daytime napping 3) and cognitive function at 2013 (5), 2015 (17), and 2018 (24) were multiply imputed using chained equations.

For determining sensitivity complete case analyses were also performed. We furthermore attempted to demonstrate the sensitivity to change of mental intactness and episodic memory as well as the overall cognitive function by classifying cognitive scores according to baseline quartiles and providing a table with proportions in these baseline quartiles for different follow up data points.

Analyses were carried out using R version 4.3.2 and Stata version 17.0 (StataCorp, College Station, TX). A two-sided p-value less than 0.05 was considered statistically significant.

### Patient and public involvement

Patients were not involved in design, recruitment, or analysis in this study.

## Results

### Baseline characteristics

Data from a total of 1,463 participants were analyzed. Of these, 749 (51.20%) individuals were aged 45–59, 618 (42.24%) individuals were aged 60–74, and the age of 96 (6.56%) individuals was ≥75 years. There were 689 males (47.10%) and 774 females (52.90%). Other baseline characteristics and baseline characteristics of participants by overall cognitive trajectory categories are shown in Table 1.

**Table 1:** Baseline characteristics of participants and baseline characteristics of participants by the overall cognitive function scores trajectories.

Characteristic	Value (n = 1463)	Low baseline, linear declining cognitive trajectory (n = 324)	Moderate baseline, linear declining cognitive trajectory (n = 549)	High-stable cognitive trajectory (n = 590)	*p*-value
**Age, n(%)**					<0.001***
** 45~59**	749 (51.20%)	111 (34.26%)	276 (50.27%)	362 (61.36%)	
** 60~74**	618 (42.24%)	162 (50%)	243 (44.26%)	213 (36.10%)	
** ≥75**	96 (6.56%)	51 (15.74%)	30 (5.46%)	15 (2.54%)	
**Female, n(%)**	774 (52.90%)	233 (71.91%)	301 (54.83%)	240 (40.68%)	<0.001***
**Educational level, n (%)**					
** No formal education**	656 (44.84%)	278 (85.8%)	281 (51.18%)	97 (16.44%)	
** Primary school**	318 (21.74%)	32 (9.88%)	149 (27.14%)	137 (23.22%)	
** Middle school or above**	489 (33.42%)	14 (4.32%)	119 (21.68%)	356 (60.34%)	
**Smoking, n (%)**					<0.001***
** Never smoker**	897(61.31%)	233 (71.91%)	342 (62.3%)	322 (54.58%)	
** Current smoker**	157(10.73%)	21 (6.48%)	61 (11.11%)	75 (12.71%)	
** Former smoker**	409(27.96%)	70 (21.60%)	146 (26.59%)	193 (32.71%)	
**Drinking, n (%)**					<0.001***
** None**	1017 (69.51%)	48 (14.81%)	129 (23.5%)	167 (28.31%)	
** < once a month**	102 (6.97%)	15 (4.63%)	38 (6.92%)	49 (8.31%)	
** ≥once a month**	344 (23. 51%)	261 (80.56%)	382 (69.58%)	374 (63.39%)	
**Nighttime sleep, n (%)**					<0.001***
** <6 h**	433 (29.60%)	130 (40.12%)	180 (32.79%)	123 (20.85%)	
** 6–8h**	614 (41.97%)	108 (33.33%)	215 (39.16%)	291 (49.32%)	
** ≥8 h**	416 (28.43%)	86 (26.54%)	154 (28.05%)	176 (29.83%)	
**Daytime napping, n (%)**					0.013*
** 0 min**	611 (41.76%)	153 (47.22%)	240 (43.72%)	218 (36.95%)	
** 1–60 min**	612 (41.83%)	117 (36.11%)	219 (39.89%)	276 (46.78%)	
** >60 min**	240 (16.40%)	54 (16.67%)	90 (16.39%)	96 (16.27%)	
**No depressive symptoms, n (%)**	916 (58.61%)	164 (50.62%)	219 (39.89%)	164 (27.8%)	<0.001***
**Overall cognitive scores, mean ± SD**	10.86 ± 4.03	5.84 ± 2.80	10.67 ± 2.77	13.79 ± 2.54	<0.001***
**Mental intactness scores, mean ± SD**	7.36 ± 3.06	3.69 ± 2.24	7.36 ± 2.42	9.39 ± 1.87	<0.001***
**Episodic memory scores, mean ± SD**	3.49 ± 1.71	2.16 ± 1.32	3.31 ± 1.44	4.40 ± 1.60	<0.001***

SD = Standard deviation, p-values for differences between cognitive trajectory groups were derived from Chi-squared tests for categorical variables, and ANOVA for continuous variables

### Estimated cognitive function trajectories

Among three alternative growth curve models (GCMs), the linear growth model, encompassing both intercept and slope, outperformed both no growth model and nonlinear growth model across overall cognitive function (TLI = 0.996, CFI = 0.997, RMSEA = 0.034), mental intactness (TLI = 0.987, CFI = 0.989, RMSEA = 0.059), and episodic memory (TLI = 0.998, CFI = 0.998, RMSEA = 0.019). Consequently, polynomial function forms, integrating intercept and slope, were adopted for the following analyses.

We assessed models with a variable number of classes, ranging from 1 to 5, using various fit statistics as detailed in S1–S3 Tables. For all three scores, the AIC, BIC, and saBIC demonstrated a consistent decrease with an increasing number of classes, suggesting a preference for a larger number. Additionally, significant p-value of ALRT indicated that a higher number of classes might yield better performance. However, it is noteworthy that as the number of classes increased, entropy exhibited a decreasing trend, implying that a smaller number of classes might yield superior results. To determine the optimal number of classes, we analyzed elbow plots (S1–S3 Figs), revealing a point of inflection at 3 classes. Hence, our comprehensive evaluation considering information-based fit indices and entropy, led us to identify three distinct trajectories for overall cognition, metal intactness, and episodic memory scores.

**Fig 2:**
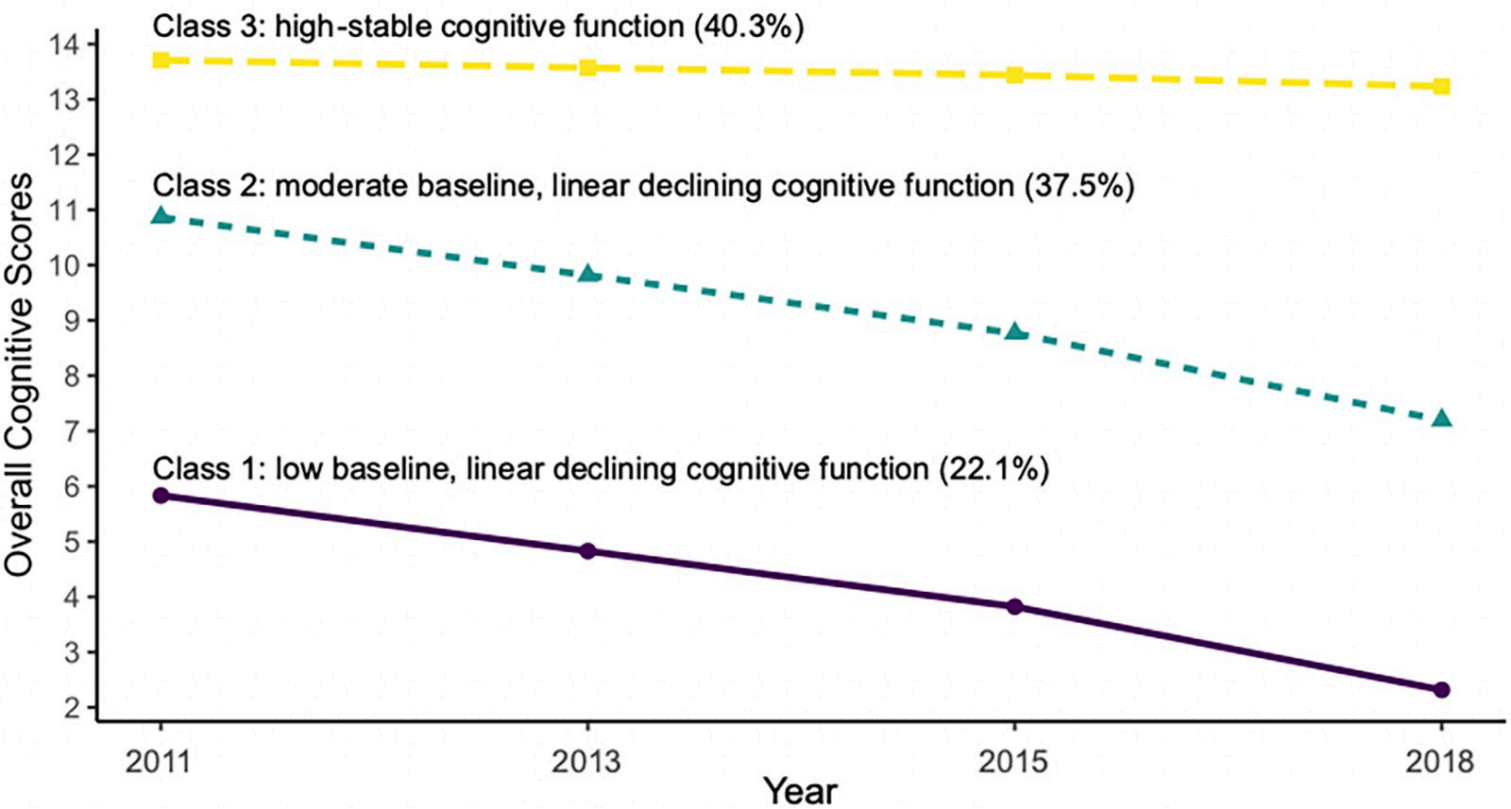
Trajectories of the overall cognitive scores by increasing age among Chinese middle-aged and older adults.

**Fig 3:**
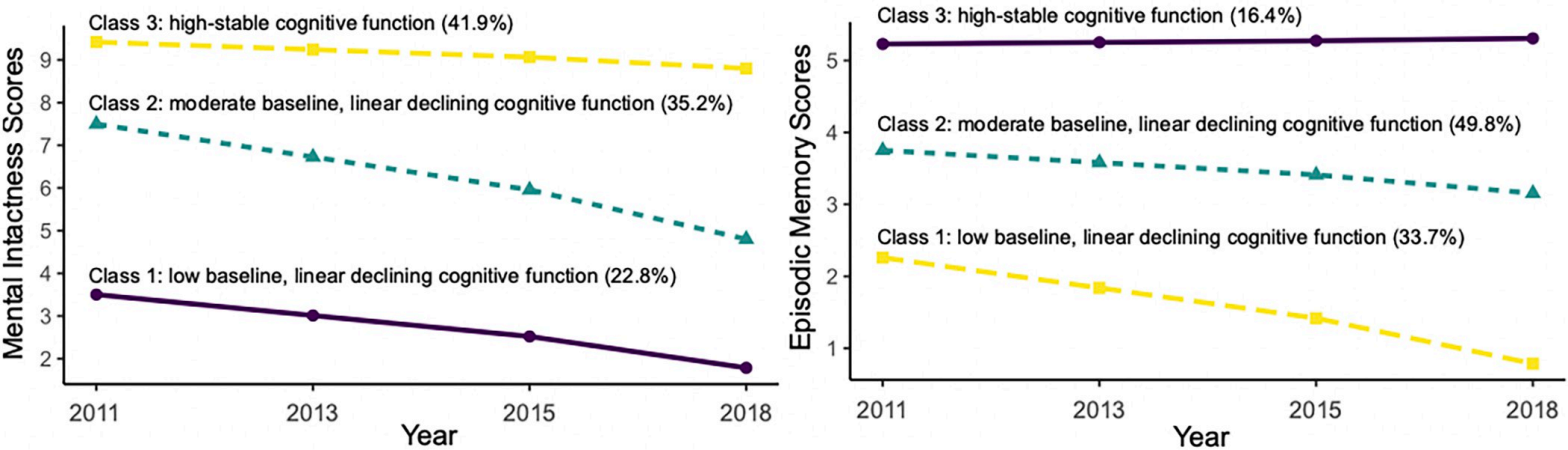
Trajectories of mental intactness scores (left) and episodic memory scores (right) by increasing age among Chinese middle-aged and older adults.

Trajectories for overall cognitive function scores are presented in Fig 2 and designated as follows group 1, “low baseline, linear declining” (22.1%); group 2, “moderate baseline, linear declining” (37.5%); and group 3, “high-stable” (40.3%). Maximum likelihood estimates for the final trajectory model of the overall cognitive function scores are shown in Table 2. Model with three trajectories also showed the best fit for mental intactness and episodic memory scores (S2 and S3 Tables). Trajectories for mental intactness and episodic memory are presented in Fig 3. Maximum likelihood estimates for the final trajectory models of mental intactness and episodic memory function scores are provided in S4 and S5 Tables.

**Table 2:** Final three-group trajectory model for development of overall cognitive scores with increasing age among Chinese middle-aged and older adults.

Trajectory group	Parameter	Maximum likelihood estimates		
		Est.	SE	*p* value
class 1, low baseline, linear decline (22.1%)	Intercept	5.833	0.160	<0.001
	Linear (age)	-0.502	0.033	<0.001
class 2, moderate baseline, linear declining (37.5%)	Intercept	10.864	0.143	<0.001
	Linear (age)	-0.524	0.030 0	<0.001
class 3, high-stable (40.3%)	Intercept	13.708	0.116	<0.001
	Linear (age)	-0.068	0.026	0.010

Est = estimate; SE = standard error

### Baseline factors of trajectories

Table 3 shows the association of baseline factors with trajectory membership as estimated with multinomial logistic regression. Older participants, females, participants with low education, with nighttime sleep <6 h, without daytime napping habits, and with depressive symptoms were likely to be assigned to more unfavorable, that is declining, overall cognitive trajectories.

**Table 3:** Multinomial logistic regression analysis of associations of risk factors with membership in the overall cognitive trajectory group.

Baseline factors	Class 2 (moderate baseline, linear declining) ref: Class 1(low baseline, linear decline)		Class 3 (high-stable) ref: Class 1 (low baseline, linear decline)		Class 3 (high-stable) ref: Class 2 (moderate baseline, linear declining)	
	*OR* (95%*CI*)	*p*-value	*OR* (95%*CI*)	*p*-value	*OR* (95%*CI*)	*p*-value
**Age(ref:45~59)**						
** 60~74**	**0.61(0.41–0.90)**	**0.011**	**0.36(0.25–0.51)**	**<0.001**	**0.59(0.44–0.81)**	**<0.001**
** ≥75**	**0.18(0.09–0.34)**	**<0.001**	**0.13(0.04–0.43)**	**<0.001**	0.72(0.20–2.53)	0.603
** Female (ref: male)**	**0.48(0.33–0.72)**	**<0.001**	**0.23(0.16–0.32)**	**<0.001**	**0.47(0.34–0.64)**	**<0.001**
**Educational level(ref: No formal education)**						
** Primary school**	**6.22(3.29–11.77)**	**<0.001**	**7.42(8.72–34.77)**	**<0.001**	**2.77(1.69–4.54)**	**<0.001**
** Middle school or above**	**4.64(2.04–10.54)**	**<0.001**	**50.36(21.64–117.21)**	**<0.001**	**10.85(6.47–18.17)**	**<0.001**
**Smoking (ref: Current smoker)**						
** Never smoker**	1.03(0.49–2.00)	0.914	0.35(0.25–0.51)	0.582	0.59(0.44–0.81)	0.551
** Former smoker**	0.72(0.34–1.54)	0.985	0.13(0.04–0.43)	0.607	0.71(0.20–2.53)	0.721
**Drinking (ref: ≥once a month)**						
** < once a month**	1.61(0.65–3.94)	0.301	1.29(0.50–3.31)	0.593	0.81(0.41–1.60)	0.544
** Never drinking**	1.26(0.69–2.32)	0.447	1.19(0.62–2.32)	0.597	0.93(0.58–1.48)	0.752
**Nighttime sleep (ref: <6 h)**						
** 6- 8h**	1.13(0.74–1.75)	0.566	**2.45(1.62–3.72)**	**<0.001**	**2.16(1.50–3.12)**	**<0.001**
** ≥8 h**	0.91(0.57–1.46)	0.703	**1.97(1.19–3.26)**	**0.009**	**2.16(1.41–3.29)**	**<0.001**
**Daytime napping (ref: 0 min)**						
** 1–60 min**	1.26(0.84–1.88)	0.263	**1.93(1.15–3.25)**	**<0.001**	**1.67(0.89–3.10)**	**0.005**
** >60 min**	1.30(0.76–2.24)	0.335	2.01(1.37–2.96)	0.112	1.60(1.15–2.22)	0.316
Depressive symptoms (ref: no depressive) symptoms)	**0.66(0.46–0.96)**	**0.028**	**0.37(0.26–0.54)**	**<0.001**	**0.56(0.41–0.78)**	**<0.001**

Ref = reference, OR = odds ratio, 95% CI = 95% confidence intervals

Risk factors related to trajectories of mental intactness and episodic memory are provided in S6 and S7 Tables. Older participants, females, participants with low education, with nighttime sleep <6 h, without daytime napping habits, and with depressive symptoms were at a higher risk of unfavorable mental intactness trajectories. Older participants, females, participants with low education, with nighttime sleep <6 h, without daytime napping habits, and with depressive symptoms were at a higher risk of unfavorable episodic memory trajectories.

### Sensitivity analyses

S8 Table reports risk factors for overall cognitive trajectories based on complete case analysis. Overall conclusions remain unchanged.

### Cognitive function scores over time

S9 Table shows that, for the overall sample and for the two groups with declining cognitive trajectories, the proportion of scores in lower baseline quartiles increases while proportions in higher baseline quartiles decrease over time. This holds true for overall cognitive scores and both mental intactness and episodic memory sub-scores, providing some evidence for sensitivity to change of the employed approach to operationalize cognitive function.

## Discussion

We analyzed cognitive function trajectories among Chinese people with diabetes aged 45 and older over an eight year period using data from the nationally representative CHARLS survey. Our study identified three overall cognitive function trajectories: a stable high cognitive function trajectory, a moderately declining, and a low-level declining trajectory. Alarmingly, a majority of people living with diabetes showed declining cognitive trajectories and about one-fifth had already low baseline cognitive function. Our study demonstrated the existence of multiple developmental cognitive function trajectories among people over 45 years old with diabetes in China, supporting the assumption that the development of cognitive function is a heterogeneous process in individuals with diabetes, rather than a homogeneous average one. While a previous study showed differential effects of diabetes on mental intactness and episodic memory scales as well as overall cognition operationalized in the same way as here [25] the present study did not find differences between trajectories for the two cognitive dimensions or overall score. This conflicting result needs to be clarified in future research. In addition, whether other dimensions of cognition have different trajectories needs further investigation.

Our study identified older people, females, and those with lower education as vulnerable populations. Modifiable risk factors included nighttime sleep <6 h, lack of daytime napping and depressive symptoms. In the Chinese context where neither pattern of cognitive function trajectories in a person with diabetes nor potential modifiable risk factors had been previously evaluated [8, 26–29]. The results of our study provide references for the design of corresponding prevention and intervention strategies. While was previously reported that cognitive impairment and dementia occur primarily in older individuals with diabetes [26], the present study found that a significant proportion of individuals aged between 45 and 59 with diabetes already shows cognitive decline. Our study is compatible with previous observations from China that females experience faster cognitive function decline than males [30, 31]. In contrast, studies from high-resource countries have not found gender differences in cognitive function [32, 33]. These differences may be related to a preference for sons in traditional Chinese society, which may be related to a higher prevalence of malnutrition in females reported since 1999 up until 2000 [34] as well as lower chances of obtaining higher education [35] as compared with male cohorts. This hypothesis needs further support from follow-up studies with cohorts born after the economic boom phase in China. We also found that participants with lower education were at higher risk of cognitive decline, this finding is in line with a previous study [36]. The relationship between education and cognition is complex. On one hand, education can improve individual cognitive function [37]. On the other hand, education may shape adult socioeconomic conditions, influencing lifestyles and access to healthcare [38]. Socially vulnerable groups including older people, females, and those with low education are at a higher risk for unfavorable trajectories. Preventive strategies should thus be designed specifically for these vulnerable groups.

Previous studies have shown that nighttime sleep patterns have an impact on cognition [39, 40]. Our study found that people with nighttime sleep <6 h were more likely to be included in the unfavorable cognitive function trajectory (low baseline, linear declining). The influence of nap time on cognition is still controversial. A cross-sectional study showed that daytime napping can reduce the risk of cognitive decline in older people over a 5 years period [41]. In contrast, a longitudinal study by Li et al showed that daytime napping was correlated with worse cognition a year later [42]. Our study found that people without napping habit were more likely to show unfavorable cognitive function trajectories (low baseline, linear decline). Our results further suggest that individuals with depression were at an increased risk of suboptimal cognitive function trajectories. Nighttime sleep, having sufficient nap time, and control of depressive symptoms may thus pose relevant intervention targets. It is worth noting here that the relationship between sleep, depression and cognition may also be influenced by other health conditions such as cardiovascular disease [43]. However, influences of comorbidity are complex and could not be fully explored in this study.

Several limitations of our study should be acknowledged. First, although the current study contributed to identifying risk factors of cognitive function among people aged 45 years and older living with diabetes in China, most risk factors were self-reported, and recall bias is unavoidable. Second, CHARLS assessment of cognition only included mental intactness and episodic memory scales. In this study, the score of overall cognitive function was calculated as the sum of the scores of mental intactness and episodic memory following previous research. Impact of diabetes and trajectories in other dimensions of cognitive function remain thus elusive. Moreover, to our knowledge no cross-calibration of subscales or overall cognitive score arrived in the manner used in this study with a gold standard such as Mini-Mental State Examination (MMSE) exists to date, precluding an analysis of clinically meaningful differences within trajectories. Our results on trajectories only reflect the cognitive function tests used in the present study, and future studies using other cognitive testing methods such as MMSE would be valuable; Third, the CHARLS data do not permit to differentiate between type 1 and type 2 diabetes, resulting in an inability to further reveal relationships between cognitive function and diabetes type in the target population. Fourth, due to data limitations, physical activity, and dietary patterns were not included in the analysis of risk factors; Fifth, progression and severity of diabetes impact cognitive function, However, data on severity and progression of diabetes were not available from the CHARLS database, so that this confounder or mediator could not be controlled for, leading to possible bias in estimates; Sixth, this holds also true for other potentially important confounders such as cardiopulmonary co-morbidity and corresponding complex interactions with cognition, diabetes, and other risk factors for cognitive decline. Finally, GMM was used for trajectory estimation in this study. It should be noted that even though grouping based on latent class facilitates data presentation and interpretation, participants do not actually belong to a single class and there will be overlap. The class membership of each participant is assigned based solely on the highest probability of belonging to one of the latent classes [44]. Caution is thus warranted when interpreting results.

In conclusion, we found that a majority of people aged 45 years and older living with diabetes in China demonstrated cognitive decline over an eight-year period, with a considerable proportion with already low baseline cognitive function. Nighttime sleep <6, absence of daytime napping habits, and depressive symptoms appear to be modifiable risk factors of cognitive decline in this population. Preventive strategies should be designed accordingly, particularly targeting vulnerable groups including females, rural residents, and those with low education.

## Supporting information

S1 Fig Elbow plot of information criteria for the overall cognitive scores group trajectories. (TIF)

S2 Fig Elbow plot of information criteria for the mental intactness scores group trajectories. (TIF)

S3 Fig Elbow plot of information criteria for the episodic memory scores group trajectories. (TIF)

S1 Table Fit statistics for the overall cognitive scores group trajectories. (DOCX)

S2 Table Fit statistics for the mental intactness scores group trajectories. (DOCX)

S3 Table Fit statistics for the episodic memory scores group trajectories. (DOCX)

S4 Table The final three-group trajectory model of mental intactness scores. (DOCX)

S5 Table The final three-group trajectory model of episodic memory scores. (DOCX)

S6 Table Multinomial logistic regression analysis for the associations of risk factors with the membership to the mental intactness scores trajectory group. (DOCX)

S7 Table Multinomial logistic regression analysis for the associations of risk factors with the membership to the episodic memory scores trajectory group. (DOCX)

S8 Table Sensitivity analyses_multinomial logistic regression analysis for the associations of risk factors with the membership to the overall cognitive function scores trajectory group. (DOCX)

S9 Table Cognitive function scores over time. (DOCX)
