# Supplementary figures and images for: Trajectories of cognitive function among people aged 45 years and older living with diabetes in China: Results from a nationally representative longitudinal study (2011~2018)
Source: PLoS One. 2024 May 24;19(5):e0299316. doi: 10.1371/journal.pone.0299316 (PMC11125531; doi:10.1371/journal.pone.0299316)

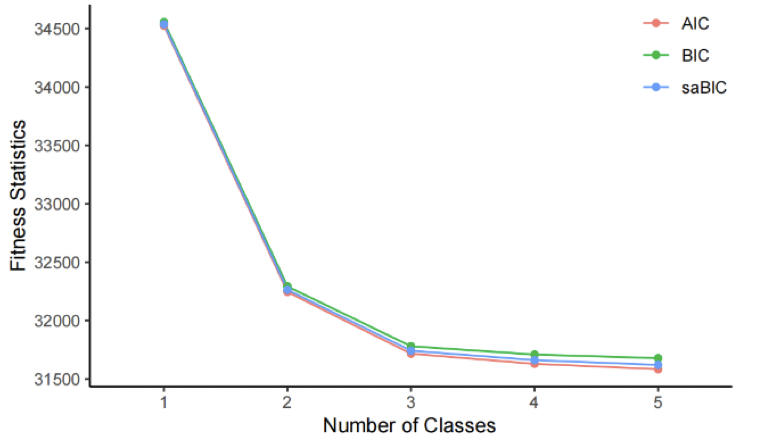

Supplement: S1 Fig — (TIF) [file pone.0299316.s001.tif]

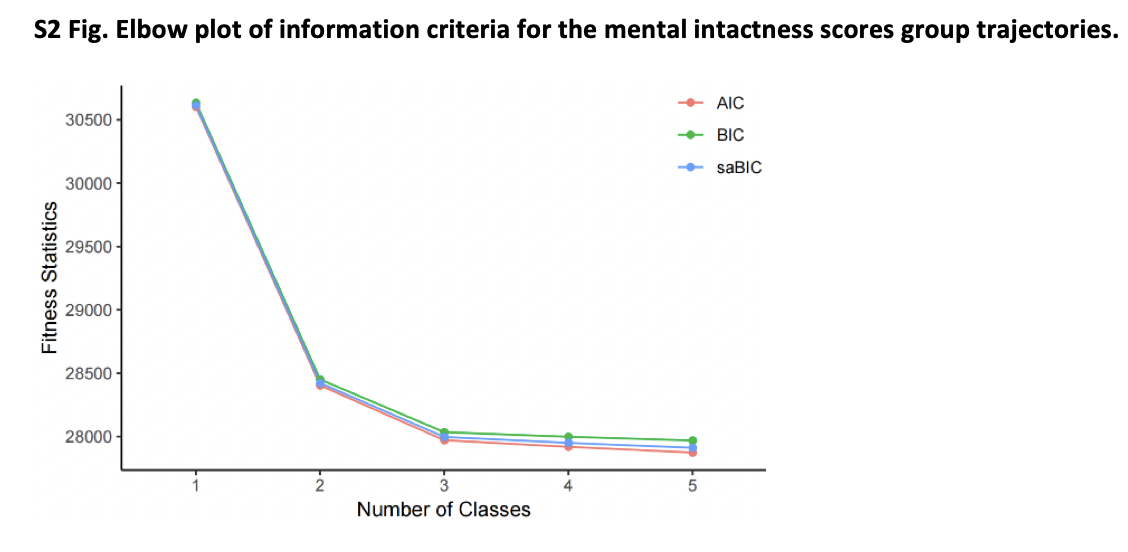

Supplement: S2 Fig — (TIF) [file pone.0299316.s002.tif]

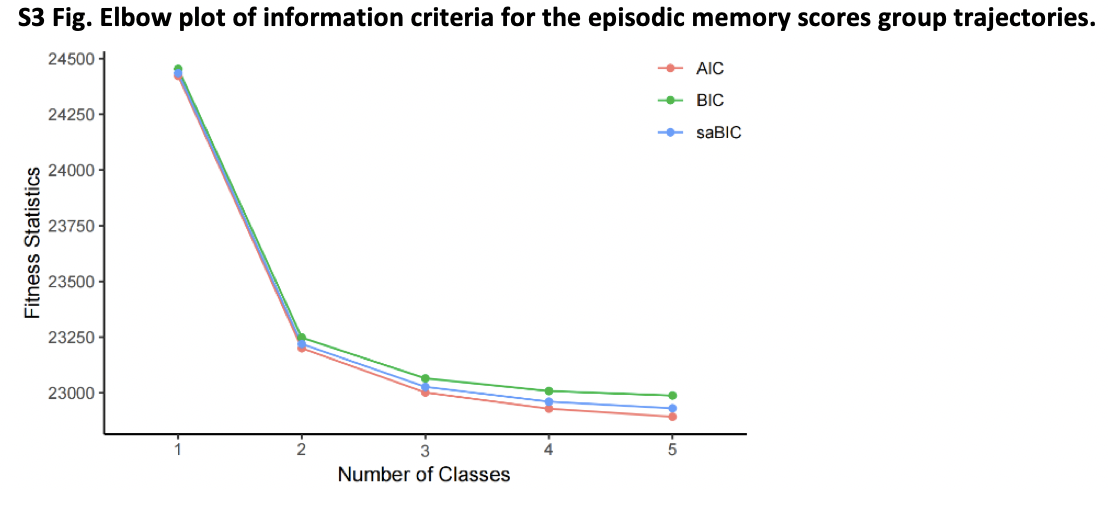

Supplement: S3 Fig — (TIF) [file pone.0299316.s003.tif]
