# Supplementary material for: Trajectories of cognitive function among people aged 45 years and older living with diabetes in China: Results from a nationally representative longitudinal study (2011~2018)
Source: PLoS One. 2024 May 24;19(5):e0299316. doi: 10.1371/journal.pone.0299316 (PMC11125531; doi:10.1371/journal.pone.0299316)
Supplement: S1 Table — (DOCX) [file pone.0299316.s004.docx]

**S1 Table. Fit statistics for the overall cognitive scores group trajectories.**

| Number of Classes | LL | AIC | BIC | saBIC | Entropy | ALRT |
| --- | --- | --- | --- | --- | --- | --- |
| 1 | -17256.34 | 34524.68 | 34556.41 | 34537.35 |  |  |
| 2 | -16113.21 | 32244.43 | 32292.02 | 32263.43 | 0.854 | <0.001 |
| 3 | -15846.15 | 31716.30 | 31779.75 | 31741.63 | 0.794 | <0.001 |
| 4 | -15799.97 | 31629.94 | 31709.27 | 31661.62 | 0.733 | <0.001 |
| 5 | -15773.46 | 31582.93 | 31678.11 | 31620.93 | 0.716 | <0.001 |

LL = Likelihood, AIC = Akaike Information Criterion, BIC = Bayesian Information Criterion, saBIC = sample-size-adjusted BIC, ALRT = Lo-Mendell-Rubin adjusted likelihood ratio test
