# Supplementary material for: Trajectories of cognitive function among people aged 45 years and older living with diabetes in China: Results from a nationally representative longitudinal study (2011~2018)
Source: PLoS One. 2024 May 24;19(5):e0299316. doi: 10.1371/journal.pone.0299316 (PMC11125531; doi:10.1371/journal.pone.0299316)
Supplement: S2 Table — (DOCX) [file pone.0299316.s005.docx]

**S2 Table. Fit statistics for the mental intactness scores group trajectories.**

| Number of Classes | LL | AIC | BIC | saBIC | Entropy | ALRT |
| --- | --- | --- | --- | --- | --- | --- |
| 1 | -15294.84 | 30601.67 | 30633.40 | 30614.34 |  |  |
| 2 | -14192.64 | 28403.29 | 28450.88 | 28422.29 | 0.856 | <0.001 |
| 3 | -13973.76 | 27971.53 | 28034.99 | 27996.86 | 0.785 | <0.001 |
| 4 | -13944.67 | 27919.34 | 27998.66 | 27951.01 | 0.727 | <0.001 |
| 5 | -13918.89 | 27873.78 | 27968.97 | 27911.79 | 0.702 | <0.001 |

LL = Likelihood, AIC = Akaike Information Criterion, BIC = Bayesian Information Criterion, saBIC = sample-size-adjusted BIC, ALRT = Lo-Mendell-Rubin adjusted likelihood ratio test
