# Supplementary material for: Trajectories of cognitive function among people aged 45 years and older living with diabetes in China: Results from a nationally representative longitudinal study (2011~2018)
Source: PLoS One. 2024 May 24;19(5):e0299316. doi: 10.1371/journal.pone.0299316 (PMC11125531; doi:10.1371/journal.pone.0299316)
Supplement: S3 Table — (DOCX) [file pone.0299316.s006.docx]

**S3 Table. Fit statistics for the episodic memory scores group trajectories.**

| Number of Classes | LL | AIC | BIC | saBIC | Entropy | ALRT |
| --- | --- | --- | --- | --- | --- | --- |
| 1 | -12205.01 | 24422.02 | 24453.75 | 24434.69 |  |  |
| 2 | -11591.12 | 23200.24 | 23247.83 | 23219.24 | 0.732 | <0.001 |
| 3 | -11488.65 | 23001.30 | 23064.76 | 23026.64 | 0.679 | <0.001 |
| 4 | -11449.33 | 22928.67 | 23007.99 | 22960.34 | 0.646 | <0.001 |
| 5 | -11428.32 | 22892.65 | 22987.84 | 22930.66 | 0.676 | <0.001 |

LL = Likelihood, AIC = Akaike Information Criterion, BIC = Bayesian Information Criterion, saBIC = sample-size-adjusted BIC, ALRT = Lo-Mendell-Rubin adjusted likelihood ratio test
