# Supplementary material for: Trajectories of cognitive function among people aged 45 years and older living with diabetes in China: Results from a nationally representative longitudinal study (2011~2018)
Source: PLoS One. 2024 May 24;19(5):e0299316. doi: 10.1371/journal.pone.0299316 (PMC11125531; doi:10.1371/journal.pone.0299316)
Supplement: S4 Table — (DOCX) [file pone.0299316.s007.docx]

**S4 Table.** **The final three-group trajectory model of** **mental intactness scores.**

| Trajectory group | Parameter | Maximum likelihood estimates | | |
| --- | --- | --- | --- | --- |
|  |  | Est. | SE | *p* value |
| class 1, low baseline, linear decline (22.8%) | Intercept | 3.504 | 0.123 | <0.001 |
|  | Linear (age) | -0.245 | 0.024 | <0.001 |
| class 2, moderate baseline, linear declining (35.2%) | Intercept | 7.497 | 0.122 | <0.001 |
|  | Linear (age) | -0.385 | 0.021  00.08 | <0.001 |
| class 3, high-stable (41.9%) | Intercept | 9.419 | 0.080 | <0.001 |
|  | Linear (age) | -0.088 | 0.019 | <0.001 |

Est = estimate; SE = standard error
