# Supplementary material for: Trajectories of cognitive function among people aged 45 years and older living with diabetes in China: Results from a nationally representative longitudinal study (2011~2018)
Source: PLoS One. 2024 May 24;19(5):e0299316. doi: 10.1371/journal.pone.0299316 (PMC11125531; doi:10.1371/journal.pone.0299316)
Supplement: S5 Table — (DOCX) [file pone.0299316.s008.docx]

**S5 Table.** **The final three-group trajectory model of episodic memory scores.**

| Trajectory group | Parameter | Maximum likelihood estimates | | |
| --- | --- | --- | --- | --- |
|  |  | Est. | SE | *p* value |
| class 1, low baseline, linear decline (33.7%) | Intercept | 2.260 | 0.093 | <0.001 |
|  | Linear (age) | -0.210 | 0.016 | <0.001 |
| class 2, moderate baseline, linear declining (49.8%) | Intercept | 3.751 | 0.076 | <0.001 |
|  | Linear (age) | -0.085 | 0.019  0 | <0.001 |
| class 3, high-stable (16.4%) | Intercept | 5.229 | 0.148 | <0.001 |
|  | Linear (age) | 0.011 | 0.024 | 0.645 |

Est = estimate; SE = standard error
