# Supplementary material for: Trajectories of cognitive function among people aged 45 years and older living with diabetes in China: Results from a nationally representative longitudinal study (2011~2018)
Source: PLoS One. 2024 May 24;19(5):e0299316. doi: 10.1371/journal.pone.0299316 (PMC11125531; doi:10.1371/journal.pone.0299316)
Supplement: S6 Table — (DOCX) [file pone.0299316.s009.docx]

**S6 Table. Multinomial logistic regression analysis for the associations of risk factors with the membership to the mental intactness scores trajectory group.**

| Baseline factors | Class 2 (moderate baseline, linear declining) ref: Class 1(low baseline, linear declining) |  | Class 3 (high-stable) ref: Class 1 (low baseline, linear declining) |  | Class 3 (high-stable) ref: Class 2 (moderate baseline, linear declining) |  |
| --- | --- | --- | --- | --- | --- | --- |
|  | OR (95%CI) | *P* | OR (95%CI) | *P* |  | *P* |
| Age(ref:45~59) |  |  |  |  |  |  |
| 60~74 | **0.47(0.33-0.67)** | **<0.001** | **0.65(0.44-0.95)** | **0.028** | **0.89(0.60-1.34)** | **0.049** |
| ≥75 | **0.09(0.05-0.18)** | **<0.001** | 0.38(0.15-1.00) | 0.05 | **0.07(0.02-0.25)** | **0.008** |
| Famale (ref: male) | **0.17(0.12-0.24)** | **<0.001** | **0.47(0.29-0.75)** | **0.002** | **0.35(0.24-0.51)** | **<0.001** |
| Educational level(ref: No formal education) |  |  |  |  |  |  |
| Primary school | **17.81(9.11-34.82)** | **<0.001** | **6.21 (3.23-11.92)** | **<0.001** | **2.60 (1.61-4.18)** | **<0.001** |
| Middle school or above | **3.97(6.42-2.46)** | **<0.001** | **4.35(4.15-4.56)** | **<0.001** | **9.51(6.02-15.02)** | **<0.001** |
| Smoking (ref: Current smoker) |  |  |  |  |  |  |
| Never smoker | 1.03(0.45-2.34) | 0.947 | 0.99(0.43-2.28) | 0.975 | 0.96(0.47-1.97) | 0.910 |
| Former smoker | 0.81(0.36-1.81) | 0.609 | 0.80(0.35-1.84) | 0.468 | 0.91(0.49-1.69) | 0.764 |
| Drinking (ref: Never drinking) |  |  |  |  |  |  |
| < once a month | 1.26(0.40-3.94) | 0.691 | 1.18(0.36-3.82) | 0.784 | 0.93(0.45-1.95) | 0.856 |
| ≥once a month | 1.15(0.60-2.18) | 0.672 | 1.08(0.55-2.13) | 0.825 | 0.94(0.60-1.48) | 0.787 |
| Nighttime sleep (ref: <6 h) |  |  |  |  |  |  |
| 6- 8h | 1.06(0.70-1.64) | 0.759 | **2.24(1.48-3.41)** | **<0.001** | **2.10(1.43-3.08)** | **<0.001** |
| ≥8 h | 1.03(0.60-1.80) | 0.906 | **1.77(1.10-2.85)** | **0.020** | **1.71(1.09-2.68)** | **0.020** |
| Daytime napping (ref: 0 min) |  |  |  |  |  |  |
| 1–60 min | 1.27(0.85-1.89) | 0.245 | **2.02(1.38-2.97)** | **<0.001** | **1.60(1.13-2.26)** | **0.008** |
| >60 min | 1.57(0.85-2.90) | 0.150 | 1.43(0.84-2.45) | 0.187 | 0.92(0.58-1.45) | 0.705 |
| Depressive symptoms (ref: no depressive symptoms) | **0.65(0.45-0.94)** | **0.022** | **0.40(0.28-0.57)** | **<0.001** | **0.62(0.44-0.56)** | **0.004** |

Ref = reference, OR = odds ratio, 95% CI = 95% confidence intervals
