# Supplementary material for: Trajectories of cognitive function among people aged 45 years and older living with diabetes in China: Results from a nationally representative longitudinal study (2011~2018)
Source: PLoS One. 2024 May 24;19(5):e0299316. doi: 10.1371/journal.pone.0299316 (PMC11125531; doi:10.1371/journal.pone.0299316)
Supplement: S7 Table — (DOCX) [file pone.0299316.s010.docx]

**S7 Table.** **Multinomial logistic regression analysis for the associations of risk factors with the membership to the episodic memory scores trajectory group.**

| Baseline factors | Class 2 (moderate baseline, linear declining) ref: Class 1(low baseline, linear declining) |  | Class 3 (high-stable) ref: Class 1 (low baseline, linear declining) |  | Class 3 (high-stable) ref: Class 2 (moderate baseline, linear declining) |  |
| --- | --- | --- | --- | --- | --- | --- |
|  | OR (95%CI) | *P* | OR (95%CI) | *P* |  | *P* |
| Age(ref:45~59) |  |  |  |  |  |  |
| 60~74 | 0.64(0.38-1.09) | 0.101 | **0.24(0.15-0.40)** | **<0.001** | **0.44(0.33-0.58)** | **<0.001** |
| ≥75 | **0.16(0.08-0.33)** | **<0.001** | **0.07(0.01-0.36)** | **0.002** | 0.34(0.07-1.72) | 0.193 |
| Famale (ref: male) | **0.59(0.38-0.94)** | **0.025** | **0.48(0.31-0.74)** | **<0.001** | 0.81(0.58-3.1.11) | 0.191 |
| Educational level(ref: No formal education) |  |  |  |  |  |  |
| Primary school | **3.35(1.20-5.60)** | **<0.001** | **29.49(60.02-144.87)** | **<0.001** | **4.31 (2.58-7.20)** | **<0.001** |
| Middle school or above | **13.40(5.76-31.17)** | **<0.001** | **66.70(139.28-332.24)** | **<0.001** | **21.18(12.33-36.37)** | **<0.001** |
| Smoking (ref: Current smoker) |  |  |  |  |  |  |
| Never smoker | 1.10(0.47-2.60) | 0.824 | 1.78(0.62-5.13) | 0.283 | 1.55(0.75-3.18) | 0.233 |
| Former smoker | 0.91(0.39-2.09) | 0.822 | 1.17(0.41-3.31) | 0.766 | 1.17(0.61-2.26) | 0.634 |
| Drinking (ref: Never drinking) |  |  |  |  |  |  |
| < once a month | 0.89(0.28-2.84) | 0.841 | 0.84(0.25-2.76) | 0.772 | 0.92(0.41-2.07) | 0.847 |
| ≥once a month | 1.18(0.44-3.19) | 0.742 | 1.10(0.41-2.99) | 0.841 | 0.88(0.52-1.50) | 0.636 |
| Nighttime sleep (ref: <6 h) |  |  |  |  |  |  |
| 6- 8h | 1.07(0.61-1.90) | 0.812 | **2.40(1.38-4.16)** | **0.002** | **2.23(1.55-3.24)** | **<0.001** |
| ≥8 h | 0.64(0.32-1.27) | 0.202 | 1.74(0.83-3.65) | 0.140 | **2.71(1.74-4.23)** | **<0.001** |
| Daytime napping (ref: 0 min) |  |  |  |  |  |  |
| 1–60 min | 1.17(0.71-1.93) | 0.549 | **1.82(1.11-2.99)** | **0.018** | **1.56(1.10-2.22)** | **0.012** |
| >60 min | 1.02(0.52-2.00) | 0.950 | 1.75(0.89-3.87) | 0.170 | **1.71(1.03-2.84)** | **0.038** |
| Depressive symptoms(ref: no depressive symptoms) | 0.94(0.59-1.48) | 0.777 | **0.38(0.23-0.62)** | **<0.001** | **0.40(0.28-0.57)** | **<0.001** |

Ref = reference, OR = odds ratio, 95% CI = 95% confidence intervals
