# Supplementary material for: Trajectories of cognitive function among people aged 45 years and older living with diabetes in China: Results from a nationally representative longitudinal study (2011~2018)
Source: PLoS One. 2024 May 24;19(5):e0299316. doi: 10.1371/journal.pone.0299316 (PMC11125531; doi:10.1371/journal.pone.0299316)
Supplement: S8 Table — (DOCX) [file pone.0299316.s011.docx]

**S8 Table.** **Sensitivity analyses_Multinomial logistic regression analysis for the associations of risk factors with the membership to the overall cognitive function scores trajectory group.**

| Baseline factors | Class 2 (moderate baseline, linear declining) ref: Class 1(low baseline, linear declining) |  | Class 3 (high-stable) ref: Class 1 (low baseline, linear declining) |  | Class 3 (high-stable) ref: Class 2 (moderate baseline, linear declining) |  |
| --- | --- | --- | --- | --- | --- | --- |
|  | OR (95%CI) | *P* | OR (95%CI) | *P* |  | *P* |
| Age(ref:45~59) |  |  |  |  |  |  |
| 60~74 | 0.64(0.38-1.09) | 0.101 | **0.24(0.15-0.40)** | **<0.001** | **0.44(0.33-0.58)** | **<0.001** |
| ≥75 | **0.16(0.08-0.33)** | **<0.001** | **0.07(0.01-0.36)** | **0.002** | 0.34(0.07-1.72) | 0.193 |
| Famale (ref: male) | **0.59(0.38-0.94)** | **0.025** | **0.48(0.31-0.74)** | **<0.001** | 0.81(0.58-3.1.11) | 0.191 |
| Educational level (ref: No formal education) |  |  |  |  |  |  |
| Primary school | **18.37(9.47-35.61)** | **<0.001** | **6.19(3.30-11.59)** | **<0.001** | **4.31 (2.58-7.20)** | **<0.001** |
| Middle school or above | **103.33(49.98-213.61)** | **<0.001** | **6.30(2.97-11.33)** | **<0.001** | **20.81(11.21-33.73)** | **<0.001** |
| Smoking (ref: Current smoker) |  |  |  |  |  |  |
| Never smoker | 1.26(0.55-2.88) | 0.586 | 1.03(0.48-2.20) | 0.937 | 0.82(0.42-1.61) | 0.559 |
| Former smoker | 0.80(0.35-1.81) | 0.586 | 0.72(0.35-1.51) | 0.388 | 0.91(0.49-1.68) | 0.760 |
| Drinking (ref: Never drinking) |  |  |  |  |  |  |
| < once a month | 1.29(0.50-3.30) | 0.595 | 1.61(0.65-3.94) | 0.301 | 1.24(0.60-2.56) | 0.554 |
| ≥once a month | 1.18(0.61-2.30) | 0.617 | 1.27(0.69-2.32) | 0.442 | 1.07(0.66-1.74) | 0.787 |
| Nighttime sleep (ref: <6 h) |  |  |  |  |  |  |
| 6- 8h | 1.17(0.76-1.81) | 0.480 | **2.58(1.70-3.92)** | **<0.001** | **2.20(1.52-3.19)** | **<0.001** |
| ≥8 h | 0.93(0.58-1.49) | 0.751 | **2.06(1.24-3.43)** | **0.005** | **2.23(1.46-3.40)** | **<0.001** |
| Daytime napping (ref: 0 min) |  |  |  |  |  |  |
| 1–60 min | 1.23(0.82-1.85) | 0.308 | **2.01(1.37-2.96)** | **<0.001** | **1.63(1.17-2.26)** | **0.004** |
| >60 min | 1.29(0.75-2.23) | 0.353 | 1.66(0.89-3.10) | 0.112 | 1.28(0.80-2.05) | 0.299 |
| Depressive symptoms(ref: no depressive symptoms) | 0.94(0.59-1.48) | 0.777 | **0.38(0.23-0.62)** | **<0.001** | **0.40(0.28-0.57)** | **<0.001** |

Ref = reference, OR = odds ratio, 95% CI = 95% confidence interval
