# Supplementary material for: Trajectories of cognitive function among people aged 45 years and older living with diabetes in China: Results from a nationally representative longitudinal study (2011~2018)
Source: PLoS One. 2024 May 24;19(5):e0299316. doi: 10.1371/journal.pone.0299316 (PMC11125531; doi:10.1371/journal.pone.0299316)
Supplement: S9 Table — (DOCX) [file pone.0299316.s012.docx]

**S9 Table.** **Cognitive function scores over time.**

|  |  | 2011 | 2013 | 2015 | 2018 | Cohen's F (95% CIs) |
| --- | --- | --- | --- | --- | --- | --- |
| Overall cognitive function scores | | |  |  |  | 1.12 [1.05, 1.19] |
| Low baseline, linear declining (n=324) | 0~8 | 266 (82.1%) | 294 (90.74%) | 303 (93.52%) | 321 (99.07%) |  |
|  | 8~11 | 46 (14.2%) | 29 (8.95%) | 19 (5.86%) | 3 (0.93%) |  |
|  | 11~14 | 11 (3.4%) | 1 (0.31%) | 2 (0.62%) | 0 (0%) |  |
|  | >14 | 1 (0.31%) | 0 (0%) | 0 (0%) | 0 (0%) |  |
| Moderate baseline, linear declining (n=549) | 0~8 | 111 (20.22%) | 161 (29.33%) | 200 (36.43%) | 359 (65.39%) |  |
|  | 8~11 | 219 (39.89%) | 195 (35.52%) | 230 (41.89%) | 134 (24.41%) |  |
|  | 11~14 | 162 (29.51%) | 145 (26.41%) | 107 (19.49%) | 48 (8.74%) |  |
|  | >14 | 57 (10.38%) | 48 (8.74%) | 12 (2.19%) | 8 (1.46%) |  |
| High-stable cognitive (n=590) | 0~8 | 11 (1.86%) | 13 (2.2%) | 10 (1.69%) | 21 (3.56%) |  |
|  | 8~11 | 84 (14.24%) | 97 (16.44%) | 95 (16.1%) | 115 (19.49%) |  |
|  | 11~14 | 228 (38.64%) | 226 (38.31%) | 241 (40.85%) | 225 (38.14%) |  |
|  | >14 | 267 (45.25%) | 254 (43.05%) | 244 (41.36%) | 229 (38.81%) |  |
| Episodic memory scores | |  |  |  |  | 0.81 [0.66, 0.87] |
| Low baseline, linear declining (n=494) | 0~2 | 270 (54.66%) | 338 (68.42%) | 376 (76.11%) | 441 (89.27%) |  |
|  | 2~4 | 185 (37.45%) | 137 (27.73%) | 113 (22.87%) | 45 (9.11%) |  |
|  | 4~5 | 31 (6.28%) | 16 (3.24%) | 4 (0.81%) | 7 (1.42%) |  |
|  | >5 | 8 (1.62%) | 3 (0.61%) | 1 (0.2%) | 1 (0.2%) |  |
| Moderate baseline, linear declining (n=729) | 0~2 | 102 (13.99%) | 103 (14.13%) | 133 (18.24%) | 212 (29.08%) |  |
|  | 2~4 | 386 (52.95%) | 378 (51.85%) | 418 (57.34%) | 286 (39.23%) |  |
|  | 4~5 | 162 (22.22%) | 168 (23.05%) | 124 (17.01%) | 139 (19.07%) |  |
|  | >5 | 79 (10.84%) | 80 (10.97%) | 54 (7.41%) | 92 (12.62%) |  |
| High-stable cognitive (n=240) | 0~2 | 2 (0.83%) | 2 (0.83%) | 2 (0.83%) | 7 (2.92%) |  |
|  | 2~4 | 42 (17.5%) | 36 (15%) | 46 (19.17%) | 32 (13.33%) |  |
|  | 4~5 | 68 (28.33%) | 66 (27.5%) | 66 (27.5%) | 64 (26.67%) |  |
|  | >5 | 128 (53.33%) | 136 (56.67%) | 126 (52.5%) | 137 (57.08%) |  |
| Mental intactness scores | |  |  |  |  | 1.10 [0.89, 1.18] |
| Low baseline, linear declining (n=335) | 0~5 | 286 (85.37%) | 304 (90.75%) | 308 (91.94%) | 332 (99.1%) |  |
|  | 5~8 | 41 (12.24%) | 30 (8.96%) | 26 (7.76%) | 3 (0.9%) |  |
|  | 8~10 | 6 (1.79%) | 1 (0.3%) | 1 (0.3%) | 0 (0%) |  |
|  | >10 | 2 (0.6%) | 0 (0%) | 0 (0%) | 0 (0%) |  |
| Moderate baseline, linear declining (n=515) | 0~5 | 113 (21.94%) | 145 (28.16%) | 171 (33.2%) | 352 (68.35%) |  |
|  | 5~8 | 231 (44.85%) | 242 (46.99%) | 251 (48.74%) | 144 (27.96%) |  |
|  | 8~10 | 127 (24.66%) | 89 (17.28%) | 84 (16.31%) | 18 (3.5%) |  |
|  | >10 | 44 (8.54%) | 39 (7.57%) | 9 (1.75%) | 1 (0.19%) |  |
| High-stable cognitive (n=613) | 0~5 | 18 (2.94%) | 26 (4.24%) | 18 (2.94%) | 25 (4.08%) |  |
|  | 5~8 | 138 (22.51%) | 169 (27.57%) | 169 (27.57%) | 213 (34.75%) |  |
|  | 8~10 | 199 (32.46%) | 209 (34.09%) | 199 (32.46%) | 235 (38.34%) |  |
|  | >10 | 258 (42.09%) | 209 (34.09%) | 227 (37.03%) | 140 (22.84%) |  |
